# Comparison of three dimensional reconstruction and conventional computer tomography angiography in patients undergoing zero-ischemia laparoscopic partial nephrectomy

**DOI:** 10.1186/s12880-020-00445-8

**Published:** 2020-05-06

**Authors:** Xiaorong Wu, Chen Jiang, Guangyu Wu, Chao Shen, Qibo Fu, Yonghui Chen, Dongming Liu, Wei Xue

**Affiliations:** 1 Department of Urology, Shanghai Jiao Tong University School of Medicine affiliated Ren Ji Hospital, 160 Pu Jian Road, 200127 Shanghai, People’s Republic of China; 2 Department of Radiology, Shanghai Jiao Tong University School of Medicine affiliated Ren Ji Hospital, 160 Pu Jian Road, 200127 Shanghai, People’s Republic of China

**Keywords:** Computer tomography angiography, Laparoscopy, Partial nephrectomy, Three-dimensional reconstruction, Zero ischemia

## Abstract

**Background:**

With the development of three dimensional (3D) reconstruction and printing technology, it has been widely using in the field of urology. However, there have been few studies reporting the role of 3D reconstruction in zero-ischemia partial nephrectomy (PN). The aim of this study was to assess the role of 3D reconstruction and conventional computer tomography angiography (CTA) in zero-ischemia laparoscopic partial nephrectomy (LPN).

**Methods:**

A total of 60 consecutive patients undergoing zero-ischemia LPN between October 2017 and March 2018 who underwent CTA (CTA group including 30 patients) and 3D reconstruction (3D group including the remaining 30 patients) preoperatively were included. 3D reconstruction and CTA images were prepared which were used to demonstrate the number and spatial interrelationships of the location of renal tumors and tumor feeding arteries. These radiological findings were directly correlated with intraoperative surgical findings at laparoscopy. Baseline, perioperative variables and the rate of accurate tumor feeding artery orientation were compared between groups.

**Results:**

All LPNs were completed without conversion to renal hilar clamping or open surgery. Preoperative 3D reconstruction identified that 15 patients had only one tumor feeding artery, 12 had two, and another 3 had three, while the conventional CTA revealed that 22 patients had one tumor feeding artery, 8 had two (*P* > 0.05). The mean operation time was shorter and estimated blood loss was less in the 3D group (*P* < 0.05) and the rate of accurate tumor feeding artery dissection was higher in the 3D group (91.7%) in comparison with the CTA group (84.2%). The baseline characteristics and renal function outcomes had no statistical differences between groups.

**Conclusions:**

3D reconstruction can provide comprehensive information for the preoperative evaluation and intraoperative orientation about tumor feeding arteries that may facilitate tumor resection during zero-ischemia LPN for renal tumors.

## Background

Laparoscopic partial nephrectomy (LPN) has gained popularity as a promising minimally invasive nephron sparing option for clinical T1 renal tumors [1]. Minimizing or even eliminating warm ischemic injury was the aim for improving the functional outcomes of nephron sparing surgery. Recent advances in surgical technique now make it possible to eliminate global ischemia completely during partial nephrectomy (PN), such as zero-ischemia PN, unclamped “minimal-margin” PN and clampless techniques [2–4]. However, zero-ischemia LPN is a technically challenging operation. As such, a detailed and case-specific understanding of the particular renovascular anatomy is of great importance to surgical planning and even orientation.

At most centers, renal angiography has been widely using for preoperative evaluation of patients with renal tumors. Although helical computerized tomography (CT) could provide high quality images of the renal tumor, vasculature and collecting system as well, three dimensional (3D) reconstruction and printing technology had gained more and more popularity in urological community [5]. These image modality has been utilizing for preoperative assessing of patients undergoing PN [6, 7]. However, there have been few studies reporting the role of 3D reconstruction in zero-ischemia PN. In present study, we aimed to determine whether 3D reconstruction could be the preferred preoperative radiological examination, which can provide all necessary information for surgical planning and orientation.

## Methods

### Patients

Between October 2017 and March 2018, 60 consecutive patients who underwent zero-ischemia LPN in our hospital were retrospectively analyzed. The patients with sporadic renal tumor at clinical T1N0M0 stage were included. Patients with tumors of solitary kidney, multiple tumors or bilateral renal tumors were excluded from this study. The study was approved by the Institutional Review Board of Ren Ji Hospital affiliated to School of Medicine of Shanghai Jiao Tong University. All the patients in this study received contrast-enhanced CT with angiography preoperatively, and additional 3D reconstruction was conducted in 30 patients.

### CTA and 3D reconstruction protocol

Before the examination of CTA, all patients were administered with one liter of water and then a 64-multidetector computed tomography scanner (VCT Light Speed, GE Healthcare, Pittsburgh, USA) was utilized to perform CTA. Non-enhanced, arterial and portal phases’ images were obtained in a craniocaudal direction covered the area from the diaphragm to the lower kidney poles, and delayed nephrographic excretory phase’s images were also obtained which covered the area from the diaphragm to the symphysis pubis. Contrast-enhanced images were gained after the administration of 150 ml of non-ionic contrast medium (Iopamiro, Bracco, Milan, Italy). The slice thickness was 1.25-mm, the pitch was 1.375 and the current was 110–380 mA. Non-enhanced nephrographic and excretory phases’ images were reconstructed as 1.25-mm sections and the arterial phase’ images were reconstructed at a 0.725-mm interval.

**Fig. 1:**
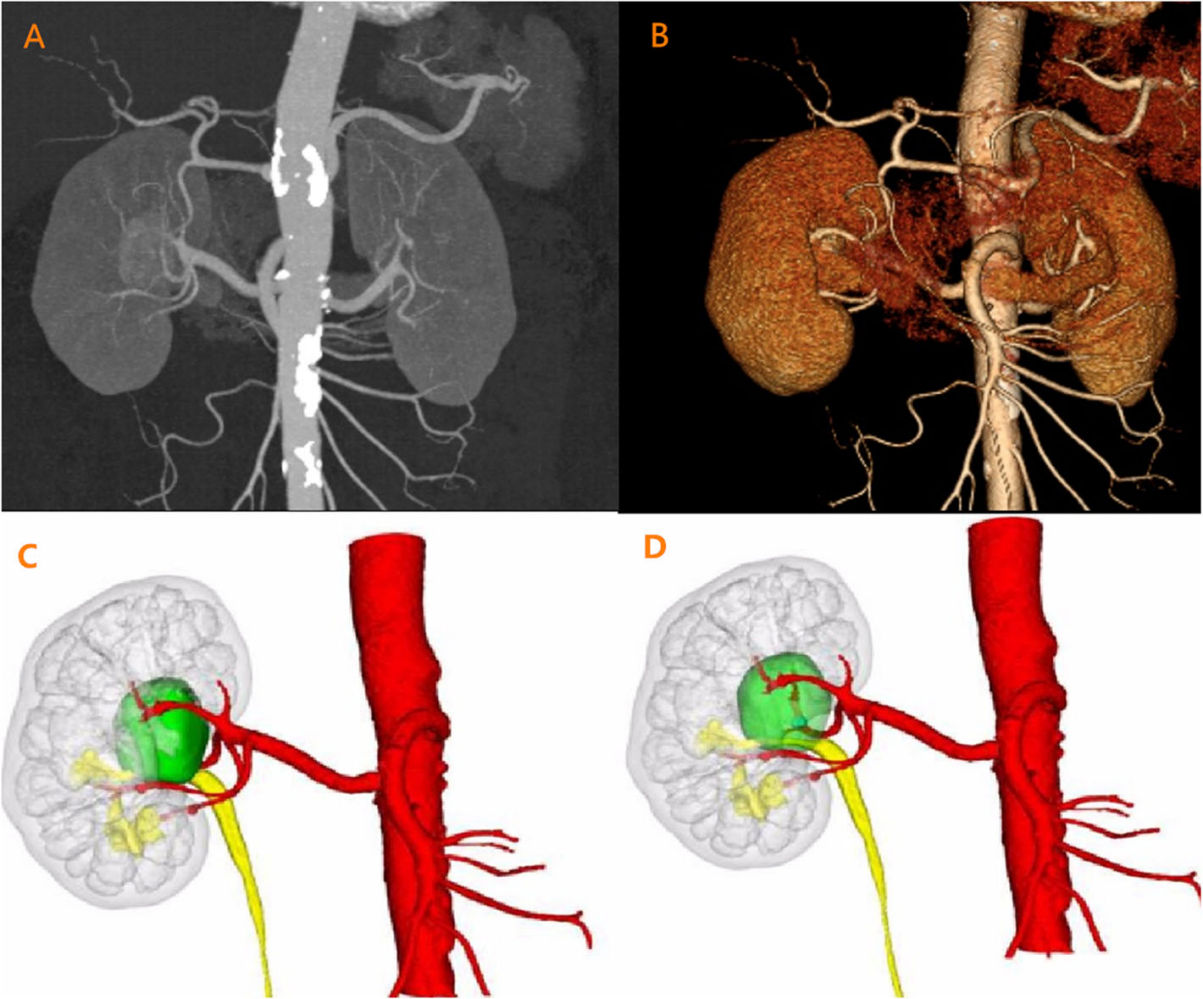
Conventional CTA and 3D reconstruction demonstrated a tumor located at the renal hilum. **a** Conventional CTA image could identify extrarenal arteries and the tumor, and sometimes the intrarenal artery branches, but failed to illustrate the relationship between tumor and its feeding arteries. **b** Conventional 3D reconstruction based on the CTA could reveal the relationship between kidney, tumor and extrarenal arteries, but it typically present kidney, tumor and renal vessels as opaque, which makes it impossible to visualize the intrarenal anatomy and tumor feeding arteries. **c**-**d** The 3D reconstruction images in our study included transparent kidney and 3D course of extra- and intrarenal tumor feeding arteries (**c**) and 3D surface rendered semitransparent renal tumor, which make it possible to show the target feeding arteries all around the tumor (**d**)

Based on the CTA examination, a dedicated software by Mimics, 3-matic and Magics (Materialise, Belgium) was using to process the images in DICOM format, and a 3D virtual model was then rendered. We reconstructed 3D images to fuse the key anatomic constructions including the renal tumors, semitransparent kidney, arterial vasculature and the collecting system. The arterial phase images with automated thresholding were used to perform the segmentation of kidney surface and the dynamic region growing method was used to reconstruct the renal pedicle, as well as tumor feeding arteries. The relative interactive 3D images and models were then created. After that, the urologists, along with radiologists and bioengineers reviewed the virtual renovascular-tumor models and assessed their accuracy in comparison with the CTA images (Fig. 1). The number of renal tumor feeding arteries was compared between the 2 imaging modalities and matched with intraoperative findings on laparoscopy, which were considered the gold standard.

### Surgical technique

**Fig. 2:**
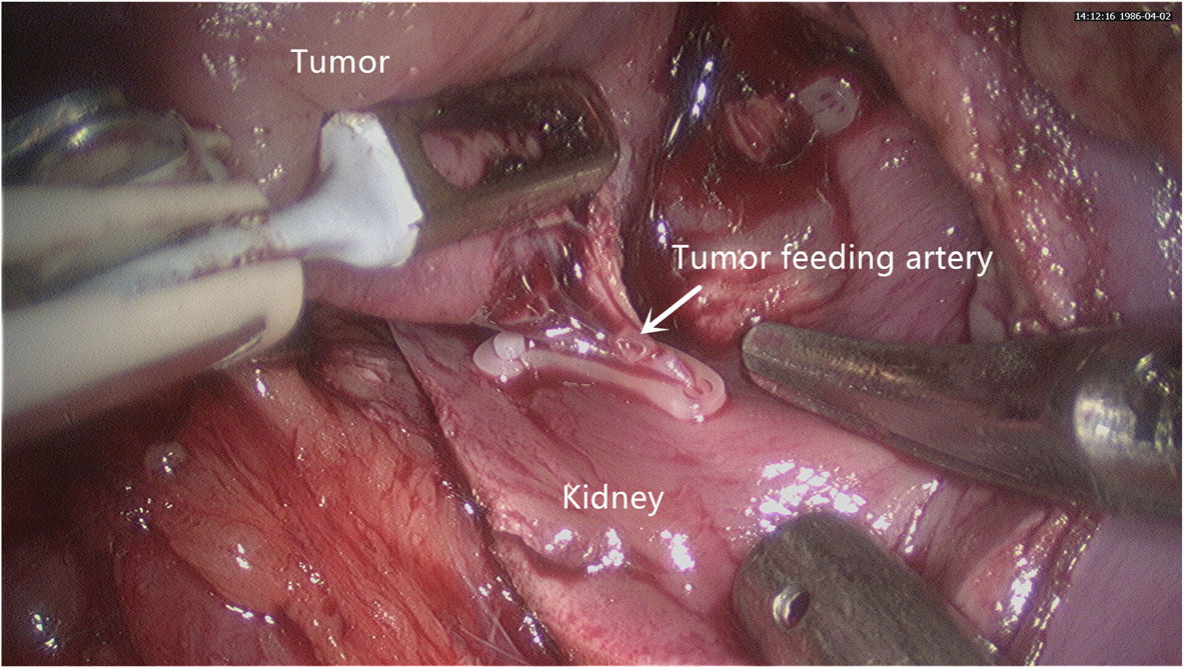
The tumor was excised with blunt dissection along its capsule. Under the guidance of 3D reconstructed images, the tumor specific feeding artery (white arrow) could be identified and then clamped with the Hem-o-lok clips during the operation

All procedures were performed by a single surgeon (D Liu). Patients were placed in a lateral decubitus position. A retroperitoneal approach was used in all operations. Zero ischemia LPN was performed under the guidance of CTA images in the CTA group, while 3D images from various angle views (e.g., anterior, posterior and lateral) were presented to the surgeon pre- and intra-operatively in the 3D group. After the Gerota’s fascia was opened, the tumor was localized, dissected and exposed completely. Then the tumor was enucleated without reanl hilar clamping by blunt and sharp dissection. The tumor feeding arteries were clamped with Hem-o-lok clips (Fig. 2) once they were found. Point-specific suturing were performed for homeostasis, and incised calyces were repaired when necessary. After then, continuous suturing with Hem-o-lok clips was used to reconstruct the defected parenchyma. A drain was left at the end of surgery and the tumor specimen were sent for pathological evaluation.

### Outcome measures

The general information, tumor characteristics including RENAL score [8], Kidney segmentation [9], intraoperative variables and the number of feeding arteries as well as the rate of accurate tumor feeding artery orientation were collected and compared. All complications within 30 days after surgery were considered being related to surgery, and staged according to the 2004 Clavien-Dindo grading system [10]. The estimated glomerular filtration rate (eGFR) was calculated for all patients preoperatively and 12 months postoperatively according to the Modification of Diet in Renal Disease Equation [11]. Follow-up was conducted at 3 and 6 months after surgery (since they were discharged) and once every 6 months thereafter. Statistical analysis was performed using Student *t*-test for continuous variables with normal distribution or Wilcoxon’s rank sum test was used for the non-normal distributed continuous variables. The comparisons of categorized demographic or clinical variables among groups were done with Pearson Chi-square or Fisher exact test, and a value of *P* < 0.05 was considered statistically significance.

## Results

A total of 60 consecutive patients who underwent zero-ischemia LPN were included in this study. Patient demographics and surgical variables are reported in Tables 1 and 2. The median tumor size was 4.0 and 3.75 cm in 3D group and CTA group respectively without statistical significance. There were also no significant differences between two groups in the age, gender, body mass index, RENAL score, and Kidney segmentation.

**Table 1:** Patients’ demographics and surgical variables

Variables	3D group (30 cases)	CTA group (30 cases)	*p* value
Gender, n (%)			0.774
Male	22 (73.3)	21 (70)	
Female	8 (26.7)	9 (30)	
Age (years, mean ± SD)	57.6 ± 11.7	56.4 ± 9.8	0.669
BMI (mean ± SD)	25.2 ± 2.8	24.9 ± 2.5	0.622
Tumor size (cm, median)	4.0	3.75	0.082
Operative time (min, mean ± SD)	125.4 ± 19.7	136.6 ± 15.1	0.017
Estimated blood loss (ml, mean ± SD)	130.3 ± 39.9	179.0 ± 77.2	0.003
Hospital stay (days, mean ± SD)	5.0 ± 1.1	5.4 ± 1.0	0.114
Pathologic subtype, n (%)			0.543
Clear cell	26 (86.7)	25 (83.3)	
Chromophobe	1 (3.3)	3 (10)	
Papillary	3 (10)	2 (6.7)	
Furhman grade, n (%)			0.740
I	10 (33.3)	12 (40)	
II	16 (53.4)	13 (43.3)	
III	4 (13.3)	5 (16.7)	
Complications, n (%)			0.365
Fever	1 (3.3%)	0	
Hematuria	1 (3.3)	3 (10%)	
eGFR change (ml/min/1.73m^2^, mean ± SD)	−8.67 ± 8.8	−10.6 ± 11.5	0.459

**Table 2:** Tumor characteristics of the patients

Variables	3D group	CTA group	*p* value
Renal Score, n (%)			0.339
4–6	11 (36.6)	12 (40)	
7–9	17 (56.7)	15 (50)	
10–12	2 (6.7)	3 (10)	
Kidney S system, n (%) No. of segments			0.949
1	4 (13.3)	5 (16.6)	
2	15 (50)	14 (46.7)	
3	9 (30)	8 (26.7)	
4	2 (6.7)	3 (10)	
Segments location			0.914
Lateral	10 (33.3)	10 (33.3)	
Medial	6 (20)	6 (20)	
Polar	10 (33.3)	11 (36.7)	
Middle	4 (13.3)	3 (10)	

The number of tumor feeding arteries was identified preoperatively by 3D reconstruction and CTA, and then confirmed intraoperatively (Table 3). According to the radiological evaluation before surgery, there were 15 patients had only one tumor feeding artery, 12 patients had two, and another 3 had three in the 3D group, while 22 patients had one, 8 patients had two in the CTA group, without significant statistical difference between groups. During the operation, more tumor feeding arteries were confirmed precisely in the 3D group than that in the CTA group CTA (*P* = 0.03). Although there was no statistical difference, the rate of precisely tumor feeding artery dissection was still higher in the 3D group (91.7%) in comparison with the CTA group (84.2%).

**Table 3:** Tumor feeding arteries according to 3D reconstruction of renovascular-tumor, conventional CTA and surgically confirmed findings in 60 patients

Variables	3D group	CTA group	*p* value
Preoperative findings of tumor feeding arteries, n (%)			0.077
1	15 (50)	22 (73.3)	
2	12 (40)	8 (26.7)	
3	3 (10)	0 (0)	
No. surgically confirmed findings, n (%)			0.03
0	1 (3.3)	1 (3.3)	
1	16 (53.3)	26 (86.7)	
2	11 (36.7)	3 (10)	
3	2 (6.7)	0 (0)	
Rate of accurately tumor feeding arteries orientation	91.7% (44/48)	84.2% (32/38)	0.285

All LPNs were completed without conversion to renal hilar clamping or open surgery. The mean operation time (OT) was shorter for the 3D group patients than for the CTA group patients (125.4 ± 19.7 min versus 136.6 ± 15.1 min, *P* = 0.017). Mean estimated blood loss (EBL) was 130.3 ± 39.9 and 179.0 ± 77.2 ml in the 3D group and the CTA group, respectively, (*P* = 0.003). There was no statistical differences between the two groups on hospital stay, perioperative complications and change in mean eGFRs (Table 1).

Pathologic studies demonstrated 51 cases of clear cell renal cell carcinoma (RCC) (26 in the 3D group and 25 in the CTA group), 4 cases of chromophobe RCC (1 in the 3D group and 3 in the CTA group), and 5 cases of papillary RCC (3 in the 3D group and 2 in the CTA group). No statistical difference was observed between the two groups on pathological subtypes and Fuhrman grade. The margins of the specimen were all negative, and no renal capsule invasion occurred. No local recurrence or metastasis was found with a median follow-up duration of 18 months in both groups.

## Discussion

During conventional LPN, renal artery clamping could provide good intraoperative visualization and bleeding control, but inevitably causes warm ischemia injury [12]. Warm ischemia injury remains one of the most important factors influencing postoperative renal function during nephron sparing surgery. Several techniques have been developed to preserve better postoperative renal function, such as segmental artery clamping and thermal ablation techniques [13, 14].

The concept of zero-ischemia LPN was firstly introduced by Gill et al. [15]. They tried to eliminate global renal ischemia by meticulous microdissection of tertiary or quaternary renal arterial branches feeding the tumor, which is based on the concept of anatomical renovascular microdissection [3]. However, dissecting tumor feeding arteries from the renal hilum is technically difficult and time-consuming. Off-clamp LPN has been developing and popularizing among the urologic communities [16, 17], and it’s very important to clarify and understand the tumor and renal vascular anatomy before surgery, especially intrarenal relationships of the tumor and feeding arteries.

As many other institutions, we preferred to utilize CTA to evaluate the patients with renal tumors. With the development of technology, CTA could provide high quality images of the renal tumor, vasculature and collecting system as well at any plane [18]. It can also facilitate establishing conventional 3D models to visualize the arterial vasculature within the hilum, guiding in choosing the appropriate hilar approach and intraoperative target orientation during segmental artery clamping [19]. Nevertheless, it cannot clearly clarify the number and location of intrarenal tumor feeding arteries. Moreover, conventional 3D models based on CTA images, typically demonstrate kidney, tumor and renal vasculature as opaque, making it difficult to observe and confirm the tumor feeding arteries [6]. Therefore, it’s essential to develop new radiologic technique for more precisely evaluation of the relevant intrarenal anatomy and for better intraoperative orientation of tumor feeding arteries.

In this series, we reconstructed 3D images to fuse the key anatomic constructions including transparent kidney, semitransparent renal tumors, collecting system, and extra- and intrarenal arterial vasculature (Fig. 1c-d). Therefore, we easily identified the number and location of tumor feeding arteries, as well as the relationship between tumors and collecting system before the operation. In addition, we dissected the target tumor feeding arteries more accurately under the guidance of 3D reconstruction, thus decreasing intraoperative OT and EBL in comparison to CTA group.

When compared to conventional CTA, 3D reconstruction techniques have several advantages [20, 21]. Porpiglia et al. compared robotic partial nephrectomies performed with or without the use of hyper-accurated 3D reconstructions, concluding that it allowed for a faithful representation of the kidney arterial vasculature, which could lead to avoiding ischemia of the healthy renal remnant [20].

Similarly, Bertolo et al. compared 3D reconstructions with the standard imaging in the capability of expanding the indications to a nephron sparing surgery for very complex renal masses. More than 20% responders changed their indication from radical to partial nephrectomy after reviewing the 3D reconstruction and it might represent a significant step toward the validation of the use of 3D reconstruction for surgical planning in patients undergoing robotic kidney surgery [21].

In summary, based on our findings, 3D reconstruction could not only provide adequate information about anatomical interrelationship between tumors, renal vasculature and collecting system, but also identify the number and location of tumor feeding arteries clearly. It’s of great use for the surgeons to make preoperative evaluation and determination of appropriate surgery strategy, and to dissect the tumor feeding arteries more precisely during zero-ischemia LPN so as to avoid invisible injury to other interlobar arteries surrounding the tumors.

The present study has some limitations. The patients who received magnetic resonance imaging were not suitable for 3D reconstruction technique, which was based on enhanced CT or CTA images. The median tumor size was 4.0 and 3.75 cm in each subgroup, the technique’s efficacy and safety in the treatment of larger renal tumors remains unknown. In order to preserve better renal function and facilitate tumor resection and renorrhaphy during zero-ischemia partial nephrectomy, robotic assisted nephron sparing surgeries were recommended in the treatment of T1b and other complex renal tumors [4, 22], but we are lacking of this experience. Under the guidance of 3D reconstruction, tumor feeding arteries were identified more accurately, but only 91.7% of the tumor feeding arteries were dissected and clamped precisely, without statistical significance in comparison to the conventional CTA technique. In addition, this was a single-center retrospective study with small sample size and short-term follow-up results, thus larger sample size study and long-term follow-up outcomes would be required in near future to confirm our findings.

## Conclusions

3D reconstruction provided comprehensive information for the preoperative evaluation and intraoperative orientation and dissection of tumor-specific feeding arteries, thus facilitating zero-ischemia LPN, which is of great importance for improving the surgical outcome.

## Data Availability

All data are fully available upon reasonable request. The corresponding author should be contacted if someone wants to request the data.

## Abbreviations

3D: Three dimensional
CT: Computerized tomography
CTA: Computer tomography angiography
EBL: Estimated blood loss
eGFR: estimated glomerular filtration rate
LPN: Laparoscopic partial nephrectomy
OT: Operation time
PN: Partial nephrectomy
RCC: Renal cell carcinoma
